# Phenol/Chloroform-Free TiO_2_-Based miRNA Extraction from Cell Lysate

**DOI:** 10.3390/ijms23168848

**Published:** 2022-08-09

**Authors:** Denisa Smela, Eliska Zelinkova, Pavel Rehulka, Zuzana Bilkova, Rudolf Kupcik

**Affiliations:** 1 Department of Biological and Biochemical Sciences, Faculty of Chemical Technology, University of Pardubice, 532 10 Pardubice, Czech Republic; 2 Department of Molecular Pathology and Biology, Faculty of Military Health Sciences, University of Defence, 500 01 Hradec Kralove, Czech Republic; 3 Biomedical Research Centre, University Hospital Hradec Kralove, 500 05 Hradec Kralove, Czech Republic

**Keywords:** miRNA isolation, TiO_2_, phenol/chloroform-free, short RNA extraction

## Abstract

While microRNAs are considered as excellent biomarkers of various diseases, there are still several remaining challenges regarding their isolation. In this study, we aimed to design a novel RNA isolation method that would help to overcome those challenges. Therefore, we present a novel phenol/chloroform-free, low-cost method for miRNA extraction. Within this method, RNA is extracted from cell lysate with an isopropanol/water/NaCl system, followed by solid-phase extraction using TiO_2_ microspheres to effectively separate short RNAs from long RNA molecules. We also demonstrated the pH-dependent selectivity of TiO_2_ microspheres towards different sizes of RNA. We were able to regulate the size range of extracted RNAs with simple adjustments in binding conditions used during the solid-phase extraction.

## 1. Introduction

MicroRNAs (miRNAs) are endogenous non-coding RNA molecules 19–23 nucleotides in length that are important for the regulation of gene expression and various cellular processes [1]. They are considered promising diagnostic and prognostic biomarkers for different pathological conditions, especially for various types of cancer [2,3]. MicroRNAs are commonly isolated from tissues, cells, and a wide variety of body fluids, such as plasma, serum, urine, saliva, or tears [4,5,6]. However, their small size, high sequence homology, and extremely low concentrations in real complex samples make the analysis of miRNAs challenging [7,8]. It is valuable to separate miRNAs from the mixture of long RNA molecules, DNA, abundant proteins, lipids, and other contaminants present in the biological matrix to prevent their negative influence on subsequent analytical steps such as polymerase chain reaction (PCR), next-generation sequencing, or microarray [9].

In the last few decades, many methods for miRNA isolation have been developed from conventional DNA and RNA extraction methods, including phenol/chloroform-based extraction (e.g., TRIzol Reagent) or silica-based solid-phase extraction (e.g., mirVana or miRNeasy) [10]. Commercial sets for miRNA isolation and purification often use a helpful liquid–liquid extraction (LLE) method; however, toxic chemicals such as phenol and chloroform are utilized (e.g., all above-mentioned sets), which are hazardous to work with and can cause contamination within the final product of RNA isolation, affecting subsequent amplification or sequencing steps [11]. In such cases, optimization of the extraction protocol is necessary and improves the quality of the isolated product; however, additional extraction and washing steps are needed [12]. Solid-phase extraction methods, which are based on specific and/or non-specific interactions between the analyte and solid-phase material, offer a simple and efficient alternative for nucleic acid isolation, as well as a way of replacing harmful organic solvent in an assay [13]. The most common, silicone dioxide resin packed in a column or in the form of a membrane, utilizes adsorption of negatively charged nucleic acids on the positively charged silicone dioxide resin. This is performed in the presence of chaotropic agents, and adsorbed nucleic acids are then eluted by applying a solution with a low salt concentration [14].

Currently, new approaches using various micro- and nanomaterials are being investigated with the aim of improving the yields and purity of extracted miRNA. Such materials offer unique characteristics, e.g., a high surface-to-volume ratio and useful magnetic, optical, or biological properties. Several studies have reported advanced nanomaterials suitable for both DNA and RNA isolation, such as graphene oxide nanoplatelets, zinc oxide nanowires and nanotubes, carbon nanotubes, and magnetite nanoparticles [15,16,17,18,19]. Another prospective material is TiO_2_—a cheap and easily available material in various commercial preparations often adopted for chromatography purposes. In proteomics, TiO_2_ materials are routinely used for selective enrichment of phosphorylated peptides due to the strong interactions between the TiO_2_ surface and phosphate groups of phosphopeptides in harsh acidic environments. Our previous results demonstrated that the TiO_2_ form, structure, and surface decoration can also significantly affect the affinity and selectivity for phosphorylated molecules [20]. Similarly, the TiO_2_ surface was used for effective interaction with DNA under strong acidic conditions (pH 2) as shown by Amano et al. [21]. On the other hand, the TiO_2_-based miRNA isolation processes described by Jimenez et al. made use of self-prepared TiO_2_ nanofibers and only high concentrations of strong chaotropic salts (similar to SiO_2_ isolation protocols) without a low pH buffer system. This study demonstrated miRNA isolation from serum or cell lysate spiked with cel-mir-54 standard with 18.0% miRNA recovery. However, the described protocol was not size-selective, and a broad size range of RNA molecules under 500 bases was isolated [22]. These findings confirm that the composition of the binding buffer (molarity, pH, etc.) strongly influences the affinity of DNA or RNA of various lengths to TiO_2_ material. 

In our work, we aimed to develop an accessible and highly selective method for short RNA isolation (miRNA) and to avoid the extraction step with toxic phenol and chloroform.

## 2. Results

**Figure 1:**
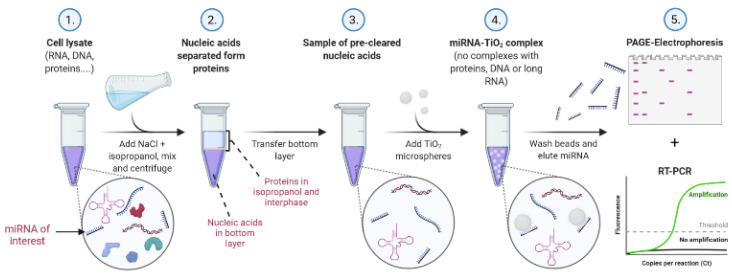
The scheme of the newly developed method for miRNA isolation from a complex biological sample. Created in Biorender.com.

Our protocol for TiO_2_-based miRNA isolation consists of a liquid–liquid extraction method using isopropanol/water/NaCl, followed by solid-phase extraction with porous 10 µm TiO_2_ microspheres under low pH in acetate, MES, or glycine buffer. The final elution of isolated miRNA is performed with Na_2_HPO_4_, which is compatible with PCR. The scheme of the experimental procedure, including specifically optimized conditions, is illustrated in Figure 1.

**Figure 2:**
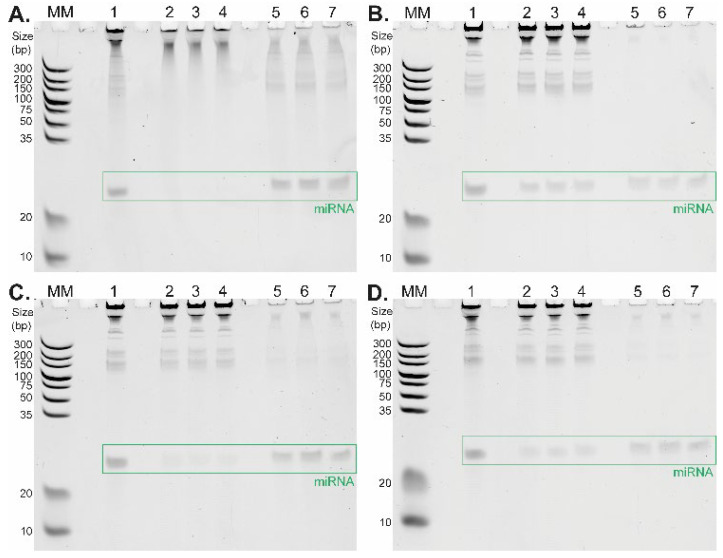
Urea-PAGE analysis of short RNA isolation (triplicate) from a model mixture (Jurkat cells genomic RNA + hsa-miR-18a-3p) in different binding conditions, followed by SYBR Green II staining. Binding conditions: (**A**) glycine/HCl buffer (pH 2.4), (**B**) MES buffer with 5 mM citric acid (pH 4), (**C**) acetate buffer (pH 4.4), and (**D**) acetate buffer with 5 mM citric acid (pH 4.4). Lanes: MM—marker of nucleotide sizes (Ultra Low Range DNA Ladder, 10 bp to 300 bp); 1—initial model mixture of RNA; 2, 3, 4—supernatant fractions; 5, 6, 7—elution fractions.

The pilot experiments showed the affinity of miRNAs towards TiO_2_ material; however, the contamination with RNAs of various sizes was observed. All protocol steps were then optimized to increase the purity of the isolated miRNA. First, we prepared a model RNA mixture consisting of genomic RNA (isolated from Jurkat cells with TRIzol Reagent) and oligo RNA standard hsa-miR-18a-3p. This mixture—containing 5.28 µg of pre-isolated genomic RNA and 0.55 µg of oligo RNA (23-mer)—was then used to optimize the binding conditions. Several washing and binding buffers within the pH range of 2.4–6 were tested for this purpose. Figure 2 depicts the differences between TiO_2_-based short RNA isolation procedures under four distinct binding conditions differing in washing and binding buffer composition tested in triplicate. The corresponding data are summarized in Table 1. The selectivity of TiO_2_ microspheres for short RNAs strongly depends on the pH of the selected buffer. The highest affinity of RNA molecules to TiO_2_ microspheres is present under strong acidic conditions (glycine/HCl buffer, pH 2.4, Figure 2A, Table 1), where a large amount of RNA molecules remains in the elution fractions (lanes 5, 6, 7). This is in line with the results of the previously reported DNA–TiO_2_ interaction study [21]. Under these conditions, the relative yields of miRNA (23-mer) determined in three replicates were 57.2%, 68.9%, and 69.6%, respectively (Table 1). Such a high yield outperformed results from the previously published study focused on TiO_2_-based miRNA isolation, where only 18.0% miRNA recovery was achieved [22]. When the pH of the washing and binding buffer was set to 4 and 4.4 (Figure 2B: MES buffer, pH 4.0; Figure 2C,D: acetate buffer, pH 4.4), long RNA molecules remained in the supernatant fractions (lanes 2, 3, 4), whereas short RNAs were extracted from the initial samples (lanes 5, 6, 7).

An interesting effect was observed with a relatively low concentration (5 mM) of citric acid in the washing and binding buffer which significantly increased the selectivity of TiO_2_ microspheres for short RNAs. Although the total miRNA yield was slightly reduced, the main advantage of this approach was the extremely high purity of miRNA in all elution fractions, as shown in Figure 2B,D, lanes 5, 6, and 7. The positive effect on miRNA isolation selectivity is even more evident when we compare the isolation of miRNA in an acetate buffer containing citric acid (relative yields were 25.4%, 30.6%, and 29.3%, Figure 2D, Table 1) with miRNA isolation in an acetate buffer without citric acid (relative yields were 28.0%, 41.0%, and 34.5%, Figure 2C, Table 1). The combination of the acetate buffer with the citric acid addition (5 mM) provides higher relative yields for miRNA isolation compared to the application of the MES buffer with the citric acid (5 mM), with corresponding relative yields of 26.0%, 18.9%, and 24.9% (Table 1). It was important to find an optimal buffer for cell lysis that would also be compatible with subsequent TiO_2_-based miRNA isolation. For this purpose, the acetate buffer system proved to be more robust and easier to work with than the MES buffer. As demonstrated, TiO_2_ microspheres successfully isolated short RNAs in the presence of all four buffer systems. In all cases, we were able to obtain high selectivity for short RNAs at higher yields compared to the previously published protocol employing neutral pH for binding conditions [22]. 

**Figure 3:**
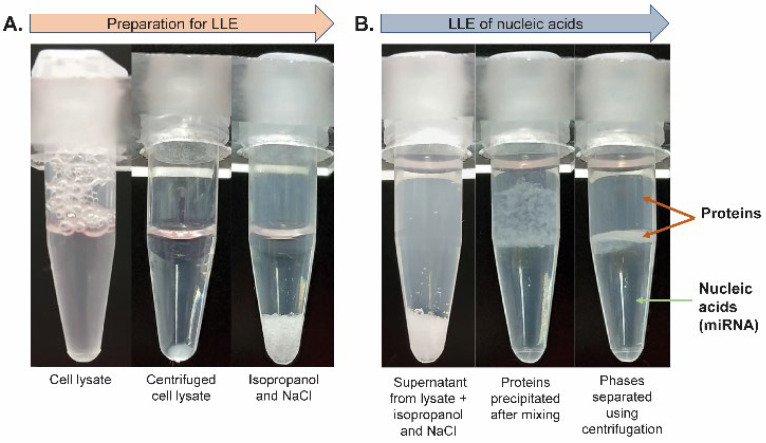
Liquid–liquid extraction of RNA from cell lysate. (**A**) Cell lysate (Jurkat cells) before and after centrifugation and tube with NaCl and isopropanol, (**B**) first tube containing isopropanol/NaCl mixed with cell lysate supernatant before incubation on a rotator, second tube after incubation step, and third tube after the final centrifugation step (top phase is organic; interphase is formed by precipitated proteins; and bottom, aqueous phase contains RNA molecules).

To accomplish the isolation of miRNA from highly complex samples such as cells, the unique phenol/chloroform-free lysis and extraction protocol was developed. All steps prior to the TiO_2_-based miRNA isolation from cells are schematically shown in Figure 3. For the evaluation of miRNA isolation effectivity and selectivity, the initial sample of Jurkat cells was spiked with oligo RNA standard (23-mer). The cells were lysed with lysis solution (100 mM sodium acetate, 2% Triton X-100, 0.1% diethyl pyrocarbonate (DEPC)). 

**Figure 4:**
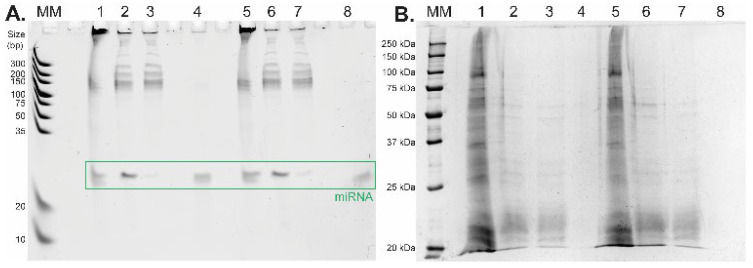
Isolation of miRNAs from cell lysate (Jurkat cells) spiked with oligo RNA standard hsa-miR-18a-3p. (**A**) Urea-PAGE analysis of extraction aliquots (duplicate) in acetate buffer pH 4.4, followed by SYBR Green II staining. MM—marker of nucleotide size (Ultra Low Range DNA Ladder, 10 bp to 300 bp), (**B**) SDS-PAGE analysis of protein purification followed by silver staining. MM—marker of molecular weight (10 to 250 kDa). Lanes: 1, 5—initial cell lysate spiked in with oligo RNA standard; 2, 6—cell lysate after LLE; 3,7—supernatant fraction; 4, 8—elution fraction.

The lysate was centrifuged (see the vial before and after centrifugation in Figure 3A) and subjected to LLE. The supernatant was transferred into a tube containing isopropanol and NaCl (see Figure 3A). The mixture was then gently rotated to gradually dissolve NaCl in acetate buffer, which led to the separation of phases, where the top phase was organic and the lower phase was aqueous (see Figure 3B). The proteins originating from the cell lysate were focused (concentrated) in the interphase after short centrifugation, as shown in Figure 3B. The bottom aqueous phase containing RNA molecules was transferred into a clean microtube and acetic acid was added to adjust the pH to ~4.4 (checked with a microelectrode-equipped pH meter). Next, the aqueous phase containing RNA was added to 1 mg of TiO_2_ microspheres for further short RNA purification and isolation applying the general isolation protocol with the acetate buffer system. The amount of oligo RNA standard and sample composition during the whole isolation process was monitored by urea-PAGE. The results of the urea-PAGE analysis of all fractions collected from the whole isolation procedure are shown in Figure 4A (the relative yields for miRNA standard were 48.1% and 68.3%, Table 2). Besides the high yield of miRNA, the main advantage of the presented results lies in the high purity of the target short non-coding RNA after its isolation from the complex mixture—see lanes 4 and 8 (Figure 4A). To show the purity of the eluted miRNAs, all collected fractions were also analyzed by Laemmli-SDS-PAGE electrophoresis with protein visualization by a silver staining method [23,24]. As shown in Figure 4B, during the LLE, most of the proteins from the cell lysate were removed from the sample (lanes 1 and 5 containing the original cell lysate vs. lanes 2 and 6 containing the bottom aqueous phase). The rest of the proteins were then removed from the solution, and the short RNA molecules were obtained by TiO_2_-based isolation during the second part of the protocol. The residual proteins are visible in the supernatant fractions after TiO_2_ miRNA purification (lanes 3 and 7), whereas no proteins were detected in the elution fractions (lanes 4 and 8).

**Figure 5:**
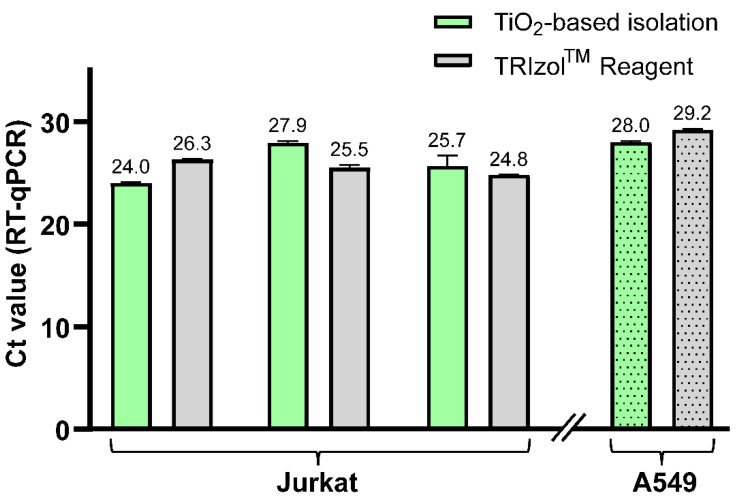
The Ct values of endogenous hsa-miR-18a-3p, obtained from RT-qPCR of elution fractions after miRNA isolation using TiO_2_-based isolation method (green) and TRIzol Reagent isolation (gray) from Jurkat cells and A549 cells (dotted pattern).

To further confirm the feasibility of this new protocol, we performed the isolation of hsa-miR-18a-3p from two types of cell lines without any added standards. Hence, we show the ability of our protocol to analyze naturally occurring miRNA in cells. The TiO_2_-based method including newly developed LLE was utilized for isolation of short RNA from both Jurkat cells (three samples) and A549 cells (one sample) and compared to RNA isolation using TRIzol Reagent. Elution fractions were analyzed by RT-qPCR in triplicate, and the results are summarized in Figure 5. The results show that we were able to isolate hsa-miR-18a-3p from both cell lines and that the newly developed TiO_2_-based method provides comparable yields to the commercial TRIzol Reagent isolation method, which is demonstrated by similar Ct values of RT-qPCR analysis. This also confirms the compatibility of the whole protocol, and mainly Na_2_HPO_4_ used for the elution of short RNA from TiO_2_ microspheres, with RT-PCR. 

## 3. Discussion

Our results from experiments using model RNA mixture shown in Figure 2 and Figure 4 clearly demonstrated the benefits of using TiO_2_ microspheres for short RNA isolation. They showed that the selective isolation of short RNA molecules is pH-dependent and the selectivity of miRNA isolation can be effectively modulated by changing the binding conditions. The isolation principle probably lies in the interaction of phosphate moieties of RNA with the TiO_2_ surface under an acidic environment. This strong interaction in combination with the porous structure of TiO_2_ particles (Titansphere, TiO) allowed selective binding of short RNA molecules, most likely due to steric hindrance of particle pores.

Moreover, the selectivity of TiO_2_ toward biomolecules containing negatively charged phosphate moieties can be modulated by hydroxy acids used as excluders, which allows us to further improve selectivity. This principle was also described for the extraction of proteins containing phosphate functional groups [25]. In our experiments, we tested several potential excluders, including citric acid, lactic acid, dihydroxybenzoic acid, glutamic acid, glycolic acid, tartaric acid, salicylic acid, 3-hydroxypicolinic acid, and sodium oxalate. However, except for citric acid (Figure 2B,D), the excluders did not have any positive effect on isolation efficiency. This could be explained by the fact that only citric acid comprises one α-position hydroxyl group and three carboxyl groups and thus contains at least seven potential donor sites capable of coordinating metal ions and can interact with TiO_2_ more strongly than other tested carboxylic acids. For applications where a high selectivity for short miRNAs is not required, the relative yields of miRNA isolation can be increased up to 41% just by omitting the citric acid from the washing and binding buffer.

For routine applications analyzing highly complex biological materials, it was necessary to adapt the protocol to extract miRNAs. Therefore, the robustness and efficiency of the protocol were tested with the whole cell lysate. For this, it was important to find an optimal buffer for cell lysis that would also be compatible with subsequent TiO_2_-based miRNA isolation. For this purpose, the acetate buffer system proved to be more robust and easier to work with than the MES buffer. The protocol was also extended for the LLE method using an isopropanol/water/NaCl system (Figure 3), followed by solid-phase extraction using TiO_2_ microspheres to effectively separate miRNAs from long RNA molecules. 

Different organic solvents (miscible and immiscible with water) were tested for optimal protein removal and miRNA isolation, including acetonitrile (ACN), isopropanol, acetone, ethyl acetate, and n-hexane. When using immiscible or partially miscible solvents, RNA purification from proteins was poor (e.g., n-hexane). Isopropanol was selected as the best solution because its application together with NaCl resulted in almost complete phase separation. This was in direct contrast with the use of acetone or ACN, which stayed partially mixed within the aqueous solution after centrifugation. The amount of NaCl is also important for a proper phase separation, because at lower concentrations (less than 4 M), the phase separation was incomplete.

For the isolation of a wider RNA size range with a higher yield, it is also possible to use a glycine buffer system. The pH of the lysis solution has to be adjusted to 6.4 using NaOH solution. After LLE, the pH of the acquired aqueous phase is lowered to ~2.4 by adding HCl before continuing with the general protocol of TiO_2_-based RNA isolation with a glycine buffer system. For effective cell lysis and LLE of RNA, it is necessary to keep the pH of the lysis solution approximately neutral, which prevents the loss of RNA molecules within the lysis procedure and exclusion from the aqueous phase into the interphase during the process of phase separation within LLE.

Our results unequivocally confirmed that this protocol combining a MES or acetate buffer containing citric acid and TiO_2_ microparticles can be applied for routine miRNA extraction from eukaryotic cells with excellent purity of the final product. This protocol could also be used for the simple separation of short RNAs from a mixture of RNAs differing in chain length, e.g., from pre-isolated genomic RNA. Glycine and acetate buffers are ideal for short RNA isolation from complex mixtures such as whole cell lysates. TiO_2_ microspheres in the environment of an acetate buffer with pH 4.4 possess high selectivity towards very short RNA molecules, resulting in extraordinary purity of miRNA isolated from such a highly complex mixture, which is clearly presented (miRNA bands in Figure 2C,D or Figure 4A). On the other hand, the glycine buffer system offers higher yields of short RNAs compared to the acetate buffer, and it allows for the isolation of a wide range of RNA lengths (Figure 2A, lanes 5, 6, 7), meaning that it can easily be used for complex RNA isolation. Furthermore, isopropanol used for LLE procedures is a common ingredient of disinfectants and can serve as an inactivation agent for bacteria or viruses. This can be advantageous when we isolate RNA molecules, e.g., from SARS-CoV-2 samples. TiO_2_-based materials are also ideal candidates for the isolation of RNA from infectious agents due to the easy decontamination of TiO_2_ surfaces by exposure to UV irradiation [26,27].

## 4. Materials and Methods

### 4.1. Microspheres and Oligo RNA

TiO_2_ microspheres, Titansphere (TiO, 10 µm) by GL Sciences (Tokyo, Japan), were used as a carrier for the solid-phase extraction.

Oligo RNA standard (23 nucleotides long) was designed analogically to the sequence of hsa-miR-18a-3p (Generi Biotech, Hradec Kralove, Czech Republic). It was used for spiking in the samples and to prepare model RNA mixture containing 5.28 µg of pre-isolated genomic RNA and 0.55 µg of oligo RNA (23-mer).

### 4.2. Cell Cultures

Jurkat cells (T-lymphoblast cell line, clone E6.1, ATCC, Manassas, VA, USA) were used for optimization of miRNA isolation from cells as well as for the preparation of a model genomic RNA. Jurkat cells cultivated in RPMI 1640 medium were harvested and washed with PBS, and a dry pellet containing 5 × 10^6^ cells was repeatedly frozen in liquid nitrogen and stored at −80 °C. RNA for model genomic RNA was isolated from Jurkat cells with TRIZol Reagent (Thermo Fisher Scientific, Waltham, MA, USA). 

A549 cells (human lung carcinoma cell line, ATCC, Manassas, VA, USA) were cultivated in MEM medium and then washed with PBS, and a dry pellet was repeatedly frozen in liquid nitrogen and stored at −80 °C.

### 4.3. General Short RNA Isolation Protocol Using TiO_2_ Microspheres

The general isolation procedure described here was applied in all isolation experiments. First, 1 mg of TiO_2_ microspheres was dispersed in 80% ACN/0.1% trifluoroacetic acid (TFA). The suspension was centrifuged at 595× *g* for 1 min to separate the solid phase from the solution after each step of sample isolation. The microspheres were washed twice with suitable washing and binding buffer to remove the ACN/TFA solution. Then, 200 µL of the initial sample in the washing and binding buffer was added and the mixture was incubated for 60 min at 37 °C. The supernatant was collected for further analysis. Weakly and non-specifically bound compounds were washed out five times with 500 µL of the washing and binding buffer (repeating each step for 2 min at 37 °C). The elution of the adsorbed miRNA was performed with 100 µL of 200 mM Na_2_HPO_4_ for 10 min at 37 °C. The elution fraction was neutralized with 5% TFA to obtain an approximately neutral pH. 

### 4.4. Short RNA Isolation from Cells

Cells (5 × 10^6^) were lysed with 260 µL of lysis solution containing 100 mM sodium acetate, 2% Triton X-100, and 0.1% diethyl pyrocarbonate (DEPC) for 10 min. The sample was spiked with 1.10 µg of oligo RNA standard. The amount of RNA standard in the fractions was monitored by urea-PAGE throughout the protocol. The lysate was centrifuged at 10,000× *g* for 10 min. An aliquot of 30 µL was recovered for further analysis, and the remaining volume (230 µL) of the cell lysate was transferred into a tube containing the same volume (230 µL) of isopropanol with 53.8 mg of NaCl. The mixture was gently rotated for 10 min to gradually dissolve NaCl in acetate buffer. This led to the protein precipitation and separation of phases, where the top phase was organic and the bottom phase was aqueous. The proteins originating from the cell lysate were concentrated in the interphase after short centrifugation at 595× *g* for 30 s. The top phase was discarded, and the bottom aqueous phase was carefully aspirated using a Hamilton syringe and transferred into a clean microtube. Acetic acid (6 M, 3.75 µL into 230 µL of aqueous phase) was added to adjust the pH to ~4.4 (checked with a microelectrode-equipped pH meter). Next, 200 µL of the aqueous phase containing RNA was added to 1 mg of TiO_2_ microspheres for further short RNA purification and isolation applying the aforementioned general isolation protocol with the acetate buffer system.

For acquiring a wider range of RNA sizes, the lysis solution consisted of 50 mM glycine, 2% Triton X-100, and 0.1% DEPC with pH adjusted to 6.4 using NaOH solution. After LLE, the pH of the acquired aqueous phase was lowered to ~2.4 by adding HCl (checked with a microelectrode-equipped pH meter) before continuing with the aforementioned general isolation protocol of TiO_2_-based RNA isolation with a glycine buffer system.

### 4.5. Polyacrylamide Gel Epectrophoresis

Polyacrylamide gel electrophoresis (PAGE) was used for the subsequent analysis of the isolated miRNAs. The aliquot of separated fractions was mixed 1:1 with a sample loading buffer (89 mM tris(hydroxymethyl)aminomethane, 89 mM boric acid, 2 mM EDTA, 7 M urea, 12% Ficoll, 0.01% xylene cyanole FF/bromphenol blue) and incubated for 4 min at 70 °C. All collected fractions were then loaded onto a 0.75 mm urea-polyacrylamide gel (20% gel with 8 M urea), and electrophoresis was performed with the Mini-Protean System (Bio-Rad, Hercules, CA, USA) at a constant voltage of 180 V in a Tris-Borate-EDTA running buffer. The gels were stained with SYBR Green II RNA Gel Stain (Thermo Fisher Scientific, Waltham, MA, USA) according to the manufacturer’s instructions. Stained gels were analyzed using the ChemiDoc XRS + System (Bio-Rad, Hercules, CA, USA), with subsequent data processing in ImageLab software (Bio-Rad, Hercules, CA, USA).

Laemmli-SDS-PAGE electrophoresis with protein visualization by a silver staining method was used for the detection of protein residue in the samples of purified RNA [23,24]. Stained gels were analyzed using the ChemiDoc XRS + System (Bio-Rad), with subsequent data processing in ImageLab software (Bio-Rad).

### 4.6. Real-Time Polymerase Chain Reaction

Elution fractions were analyzed by RT-qPCR (TaqMan miRNA assay, Thermo Fisher Scientific, Waltham, MA, USA) according to the manufacturer’s instructions. First, miRNA was reverse transcribed into cDNA using hsa-miR-18a-3p-specific RT primer. A TProfessional Basic Thermocycler (Biometra Ltd., Göttingen, Germany) was used for cDNA synthesis with the following program: 30 min at 16 °C, 30 min at 42 °C, and 5 min at 85 °C. The cDNA transcript was then used for detection by real-time quantitative PCR with TaqMan miRNA assay. The reaction was carried out on RotorGene RG-3000A (Corbett Research, Cambridge, United Kingdom) using a real-time quantitative program: 10 min at 95 °C, then 15 s at 95 °C and 60 s at 60 °C for a total of 40 cycles.

## 5. Conclusions

In conclusion, a new highly selective two-step method for miRNA extraction from cell lysates was developed without any use of toxic reagents. We showed that TiO_2_-based RNA isolation works in four buffer systems with different pHs. Moreover, in two of these (acetate or MES buffer), we were able to efficiently separate short RNAs from long RNAs, while still acquiring high miRNA yields. It was also experimentally proven that this method is compatible with a subsequent RT-qPCR analysis and achieved similar yields of native miRNA to the commercial TRIzol Reagent. Compared to conventional methods based on phenol/chloroform extraction, the proposed method provides several advantages, such as low cost (approximately USD 1 per analysis vs. approximately USD 2.7 analysis with TRIzol Reagent or approximately USD 16.8 per analysis with mirVana miRNA Isolation Kit), more environmentally friendly (green) chemistry, and most importantly, higher yield of miRNAs after extraction from real biological samples. It can be applied for routine isolation and identification of miRNAs in their analysis as a new molecular marker. Compared to traditional methods, the design and parameters of this TiO_2_-based method are better suited for the requirements of clinical practice, with an option for easy automation and miniaturization.

## Figures and Tables

**Table 1:** The relative yields of hsa-miR-18a-3p in fractions obtained during the isolation protocol using model RNA mixture in different binding conditions.

Binding Conditions	Rel. Intensity of hsa-miR-18a-3p Initial Sample (%)	Rel. Intensity of hsa-miR-18a-3p Supernatant Fractions (%)			Rel. Intensity of hsa-miR-18a-3p Elution Fractions (%)		
Glycine/HCl buffer (pH 2.4)	100	8	9	6	57.2	68.9	69.6
MES buffer + 5 mM citric acid (pH 4.0)	100	36	38	36	26.0	18.9	24.9
Acetate buffer (pH 4.4)	100	10	7	8	28.0	41.0	34.5
Acetate buffer + 5 mM citric acid (pH 4.4)	100	19	21	21	25.4	30.6	29.3

**Table 2:** The relative yields of hsa-miR-18a-3p in fractions obtained during the isolation protocol from cell lysate spiked with hsa-miR-18a-3p.

Binding Conditions	Rel. Intensity of hsa-miR-18a-3p Lysate after LLE (%)	Rel. Intensity of hsa-miR-18a-3p Supernatant Fractions (%)		Rel. Intensity of hsa-miR-18a-3p Elution Fractions (%)	
Acetate buffer (pH 4.4)	100	6	8	48.1	68.3

## Data Availability

The data presented in this study are available on request from the corresponding author.

## References

[1] Bartel D.P. (2004). MicroRNAs: Genomics, Biogenesis, Mechanism, and Function. Cell.

[2] Calin G.A., Sevignani C., Dumitru C.D., Hyslop T., Noch E., Yendamuri S., Shimizu M., Rattan S., Bullrich F., Negrini M. (2004). Human MicroRNA Genes Are Frequently Located at Fragile Sites and Genomic Regions Involved in Cancers. Proc. Natl. Acad. Sci. USA.

[3] Lu J., Getz G., Miska E.A., Alvarez-Saavedra E., Lamb J., Peck D., Sweet-Cordero A., Ebert B.L., Mak R.H., Ferrando A.A. (2005). MicroRNA Expression Profiles Classify Human Cancers. Nature.

[4] Barad O., Meiri E., Avniel A., Aharonov R., Barzilai A., Bentwich I., Einav U., Gilad S., Hurban P., Karov Y. (2004). MicroRNA Expression Detected by Oligonucleotide Microarrays: System Establishment and Expression Profiling in Human Tissues. Genome Res..

[5] Calin G.A., Liu C.-G., Sevignani C., Ferracin M., Felli N., Dumitru C.D., Shimizu M., Cimmino A., Zupo S., Dono M. (2004). MicroRNA Profiling Reveals Distinct Signatures in B Cell Chronic Lymphocytic Leukemias. Proc. Natl. Acad. Sci. USA.

[6] Weber J.A., Baxter D.H., Zhang S., Huang D.Y., Huang K.H., Lee M.J., Galas D.J., Wang K. (2010). The MicroRNA Spectrum in 12 Body Fluids. Clin. Chem..

[7] Brunet-Vega A., Pericay C., Quílez M.E., Ramírez-Lázaro M.J., Calvet X., Lario S. (2015). Variability in MicroRNA Recovery from Plasma: Comparison of Five Commercial Kits. Anal. Biochem..

[8] Ntelios D., Georgiou E., Alexouda S., Malousi A., Efthimiadis G., Tzimagiorgis G. (2022). A Critical Approach for Successful Use of Circulating MicroRNAs as Biomarkers in Cardiovascular Diseases: The Case of Hypertrophic Cardiomyopathy. Heart Fail. Rev..

[9] Dong H., Lei J., Ding L., Wen Y., Ju H., Zhang X. (2013). MicroRNA: Function, Detection, and Bioanalysis. Chem. Rev..

[10] Brown R.A.M., Epis M.R., Horsham J.L., Kabir T.D., Richardson K.L., Leedman P.J. (2018). Total RNA Extraction from Tissues for MicroRNA and Target Gene Expression Analysis: Not All Kits Are Created Equal. BMC Biotechnol..

[11] Zaporozhchenko I.A., Morozkin E.S., Skvortsova T.E., Bryzgunova O.E., Bondar A.A., Loseva E.M., Vlassov V.V., Laktionov P.P. (2015). A Phenol-Free Method for Isolation of MicroRNA from Biological Fluids. Anal. Biochem..

[12] Toni L.S., Garcia A.M., Jeffrey D.A., Jiang X., Stauffer B.L., Miyamoto S.D., Sucharov C.C. (2018). Optimization of Phenol-Chloroform RNA Extraction. MethodsX.

[13] Ali N., Rampazzo R.d.C.P., Costa A.D.T., Krieger M.A. (2017). Current Nucleic Acid Extraction Methods and Their Implications to Point-of-Care Diagnostics. Biomed Res. Int..

[14] Boom R., Sol C.J., Salimans M.M., Jansen C.L., Dillen P.M.W., Noordaa J.v.d. (1990). Rapid and Simple Method for Purification of Nucleic Acids. J. Clin. Microbiol..

[15] Hashemi E., Akhavan O., Shamsara M., Rahighi R., Esfandiar A., Tayefeh A.R. (2014). Cyto and Genotoxicities of Graphene Oxide and Reduced Graphene Oxide Sheets on Spermatozoa. RSC Adv..

[16] Park J.S., Goo N.-I., Kim D.-E. (2014). Mechanism of DNA Adsorption and Desorption on Graphene Oxide. Langmuir.

[17] Saha S., Sarkar P. (2014). Understanding the Interaction of DNA–RNA Nucleobases with Different ZnO Nanomaterials. Phys. Chem. Chem. Phys..

[18] Nandy B., Santosh M., Maiti P.K. (2012). Interaction of Nucleic Acids with Carbon Nanotubes and Dendrimers. J. Biosci..

[19] Saiyed Z.M., Bochiwal C., Gorasia H., Telang S.D., Ramchand C.N. (2006). Application of Magnetic Particles (Fe_3_O_4_) for Isolation of Genomic DNA from Mammalian Cells. Anal. Biochem..

[20] Kupcik R., Macak J.M., Rehulkova H., Sopha H., Fabrik I., Anitha V.C., Klimentova J., Murasova P., Bilkova Z., Rehulka P. (2019). Amorphous TiO_2_ Nanotubes as a Platform for Highly Selective Phosphopeptide Enrichment. ACS Omega.

[21] Amano T., Toyooka T., Ibuki Y. (2010). Preparation of DNA-Adsorbed TiO_2_ Particles—Augmentation of Performance for Environmental Purification by Increasing DNA Adsorption by External PH Regulation. Sci. Total Environ..

[22] Jimenez L.A., Gionet-Gonzales M.A., Sedano S., Carballo J.G., Mendez Y., Zhong W. (2018). Extraction of MicroRNAs from Biological Matrices with Titanium Dioxide Nanofibers. Anal. Bioanal. Chem..

[23] Laemmli U.K. (1970). Cleavage of Structural Proteins during the Assembly of the Head of Bacteriophage T4. Nature.

[24] Oakley B.R., Kirsch D.R., Morris N.R. (1980). A Simplified Ultrasensitive Silver Stain for Detecting Proteins in Polyacrylamide Gels. Anal. Biochem..

[25] Aryal U.K., Ross A.R.S. (2010). Enrichment and Analysis of Phosphopeptides under Different Experimental Conditions Using Titanium Dioxide Affinity Chromatography and Mass Spectrometry. Rapid Commun. Mass Spectrom..

[26] Wu T., Xu T., Chen Y., Yang Y., Xu L.-P., Zhang X., Wang S. (2018). Renewable Superwettable Biochip for MiRNA Detection. Sens. Actuators B Chem..

[27] Molina-Reyes J., Romero-Morán A., Sánchez-Salas J.L. (2020). Enhanced Photocatalytic Bacterial Inactivation of Atomic-Layer Deposited Anatase-TiO_2_ Thin Films on Rutile-TiO_2_ Nanotubes. Photochem. Photobiol. Sci..

